# Supplementary material for: An equation of state unifies diversity, productivity, abundance and biomass
Source: Commun Biol. 2022 Aug 25;5:874. doi: 10.1038/s42003-022-03817-8 (PMC9411111; doi:10.1038/s42003-022-03817-8)
Supplement: Supplementary file 2 — Supplemmentary Information [file 42003_2022_3817_MOESM2_ESM.pdf]

Supplementary information for

An Equation of State Unifies Diversity, Productivity, Abundance and Biomass

John Harte, Micah Brush, Erica A. Newman, Kaito Umemura

Correspondence to: jharte@berkeley.edu

This PDF file includes:

Supplementary Note 1: Pairwise correlations among state variables

Supplementary Note 2: Deriving the closed form ecological equation of state

Supplementary Note 3: Data sources and extended acknowledgements

Supplementary Note 4: Sensitivity of the accuracy of the equation of state to the metabolic scaling law

Figures S1-S4

Table S1-S2

References S1 – S20

Supplementary Note 1: Pairwise correlations among state variables

While the four-variable (*S*, *N*, *E*, *B*) ecological equation of state appears to reliably describe a variety of ecosystems (Eq. 2 and Fig. 1 of main text), the question naturally arises as to whether simpler relationships among the state variables are equally successful.

Here we examine the pairwise comparisons among the observed values, or logarithms of observed values, of the four state variables and contrast the resulting regression coefficients with the regression coefficients obtained when observed species richness is compared with the value of species richness predicted by the equation of state, or when observed logarithm of biomass is compared with the value of ln(*B*) predicted by the equation of state.

Figures S1.a-f show the six possible pairwise comparisons between the natural logarithms of the observed values of the four state variables. Regression coefficients are summarized in Table S1.

Of the six pairwise comparisons, that of ln(*B*) versus ln(*E*) has the highest R^2 value (Fig. S1.f), which is expected because *B* and *E* are computed from the same data. We also note from Figs. S1.a-c, that comparing the three possible single-state variable predictors of ln(*S*), namely ln(*N*), ln(*E*) and ln(*B*), ln(*N*) has the highest explanatory power, although there is still much scatter around the regression line.

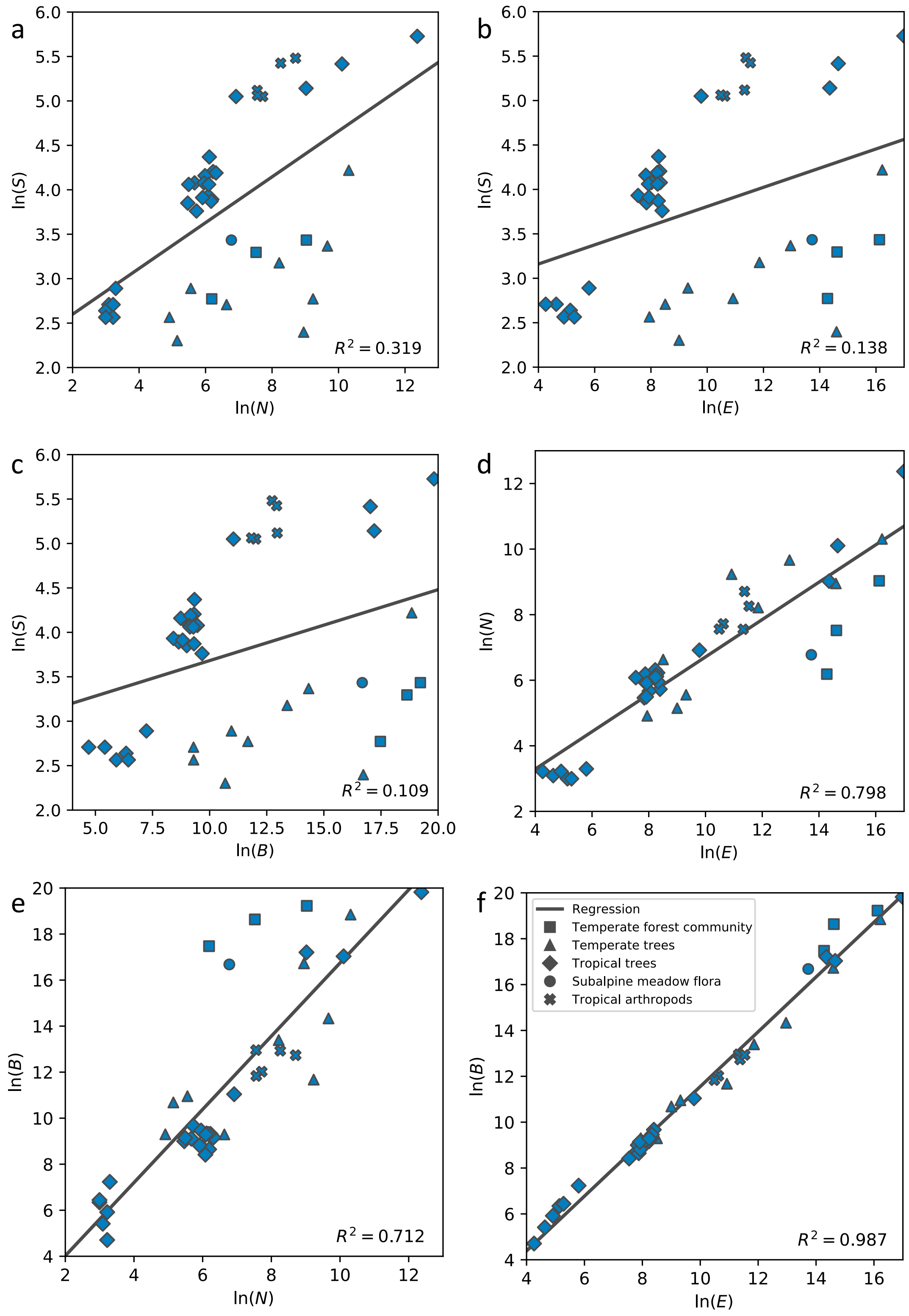

Fig. S1. The six (a, …, f) pairwise comparisons among the four state variables.

	$\ln(N)$	$\ln(E)$	$\ln(B)$
$\ln(S)$	0.319	0.138	0.109
$\ln(N)$		0.798	0.712
$\ln(E)$			0.987

Table S1. Summary of the R^2 values from pairwise comparisons among explanatory variables discussed above.

Supplementary Note 2. Deriving the closed form ecological equation of state

To derive a closed form for the equation of state, we need to find an analytic approximation for the equation for the biomass, $B = S\sum_n \int_\varepsilon n\ \varepsilon^{4/3} R(n, \varepsilon \mid S, N, E)\ d\varepsilon$. We use the methods from SI-E in ref. 23 in the main text to derive this approximation. In the notation used there, approximations to equations of the form $I = \sum_n \int_\varepsilon n^\nu\ \varepsilon^\sigma e^{-\lambda_1 n - \lambda_2 n\varepsilon}\ d\varepsilon$ are derived. The equation for *B* is of that form, with $\nu = 1$ and $\sigma = 4/3$. From Eq. E-10 in ref. S1, the leading order terms for this integral with $\sigma > \nu$ are

$$I \approx \frac{\Gamma(\sigma+1)}{{\lambda_2}^{\sigma+1}}\left(\frac{1}{\sigma-\nu} + \frac{e^{-\lambda_1}}{2} + \Gamma(\nu-\sigma)\beta^{\sigma-\nu}\right) = \frac{\Gamma\left(\frac{7}{3}\right)}{{\lambda_2}^{\frac{7}{3}}}\left(3 + \frac{e^{-\lambda_1}}{2} + \Gamma\left(-\frac{1}{3}\right)\beta^{\frac{1}{3}}\right). \qquad \text{(S1)}$$

Approximating $e^{-\lambda_1} \approx 1$ and substituting into the equation for *B* (Eq. 1 in main text),

$$B \approx \frac{4.17\ S}{Z{\lambda_2}^{\frac{7}{3}}}\left(1 - 1.16\beta^{\frac{1}{3}}\right), \qquad (S2)$$

where *Z* is the normalization and is approximately equal to ln $(1/\beta)/\lambda_2$, and λ_2 is approximately *S/E* (ref 10 in the main text). Substituting these approximations into Eq. S2 gives

$$B \approx \frac{4.17\ E^{\frac{4}{3}}}{S^{\frac{1}{3}} \ln\left(\frac{1}{\beta}\right)}\left(1 - 1.16\beta^{\frac{1}{3}}\right), \qquad \text{(S3)}$$

which is Eq. 2 in the main text, with an additional first order correction factor of $1 - 1.16\beta^{1/3}$.

To see why the integral *I* gives this result, we summarize the arguments in SI-E of ref. S1 for this specific case, with $\nu = 1$ and $\sigma = 4/3$. To make this approximation, we first take the sum and the integral to go to infinity rather than *N* and *E*, and then additionally approximate the sum over

n as an integral. The first order correction for approximating a sum as an integral is half of the value of the function being summed over evaluated at the endpoints of the sum, that is $\sum_{n=1}^{\infty} f(n) \approx \int_{n=1}^{\infty} f(n)dn + \frac{f(1)+f(\infty)}{2}$. In this case, this leads to a correction term $\int_{\varepsilon} \varepsilon^{4/3} e^{-\lambda_1 - \lambda_2 e} \, d\varepsilon/2$, since the term at infinity is negligible. With these approximations,

$$I \approx \int_{n=1}^{\infty} \int_{\varepsilon=1}^{\infty} n\, \varepsilon^{\frac{4}{3}} e^{-\lambda_1 n - \lambda_2 n \varepsilon} \, dn \, d\varepsilon + \frac{e^{-\lambda_1}}{2} \int_{\varepsilon=1}^{\infty} \varepsilon^{\frac{4}{3}} e^{-\lambda_2} d\varepsilon. \tag{S4}$$

The integral over ε in both terms can be recognized as the generalized exponential integral $E_{-4/3}(\lambda_2 n)$, with $n = 1$ in the second term. The generalized exponential integral is defined as $E_p(z) = \int_1^{\infty} e^{-zt}/t^p dt.$ In this case, we can take the first term from the expansion of this function using Eq. 8.19.10 from the Digital Library of Mathematical Functions (DLMF, ref S2) ($E_p(z) \approx z^{p-1}\Gamma(1-p)$). This means we can use $\int_{\varepsilon=1}^{\infty} \varepsilon^{4/3} e^{-\lambda_2 n e} \, d\varepsilon \approx \frac{\Gamma(7/3)}{(\lambda_2 n)^{7/3}}$. Note that this is only valid in this case because the exponent on ε is greater than that on n. This means that this approximation works well for large n, even though $\lambda_2 n$ is not small, because the subsequent integral over n has a factor of $n^{4/3}$ in the denominator, and since that power is larger than 1 that integral can be approximated again in the same way. Using the approximation of the integral over ε gives us

$$I \approx \frac{\Gamma\left(\frac{7}{3}\right)}{\lambda_2^{\frac{7}{3}}} \left(\int_{n=1}^{\infty} \frac{e^{-\lambda_1 n}}{n^{\frac{4}{3}}} dn + \frac{e^{-\lambda_1}}{2} \right). \tag{S5}$$

The integral over n is another generalized exponential integral, $E_{4/3}(\lambda_1)$, and we take the zeroth and first order terms from the same expansion as before (Eq. 8.19.10 from ref. S2). This gives us $\int_{n=1}^{\infty} \frac{e^{-\lambda_1 n}}{n^{4/3}} dn \approx \lambda_1{}^{1/3}\Gamma(-1/3) - \frac{1}{1-4/3} = 3 + \Gamma(-1/3)\lambda_1{}^{1/3}$. Plugging this in gives our final approximation of the integral,

$$I \approx \frac{\Gamma\left(\frac{7}{3}\right)}{\lambda_2^{\frac{7}{3}}} \left(3 + \frac{e^{-\lambda_1}}{2} + \Gamma\left(-\frac{1}{3}\right) \beta^{\frac{1}{3}} \right). \tag{S6}$$

Note that we have used β in place of λ_1 here as for most ranges of state variables $\lambda_1 \approx \beta$, but this also ensures that we can still use this approximation if $\lambda_1 < 0$. Note also that the more careful derivation in SI-E of ref. S1 obtains β rather than λ_1 here anyway. We can then plug this expression for *I* into the equation for *B* (Eq. 1 in the main text) to get an analytic approximation for *B*.

We then test the accuracy of this analytic expression compared to the direct numerical calculation for *B* for different values of the state variables. Fig. S2 shows direct comparisons of the analytical approximation to the numerical calculation for a wide range of *E* and *N* with *S* held fixed at 50 (see caption), and both Fig. S2**a** without the first order term (as Eq. 2 in the main text) and Fig. S2**b** with the first order correction. The contours in this plot are calculated as the negative of $\log_{10}$ of the percent difference between the analytic and numerical calculations, or $-\log_{10}\left|\frac{B_{\mathrm{approx}}-B_{\mathrm{num}}}{B_{\mathrm{num}}}\right|$, where B_{approx} is the biomass calculated using the analytic approximation, and B_{num} is calculated numerically. This means that the different contours correspond to the number of decimal places the approximation is good to. For example, the contour of level 1 corresponds to a 10% difference between the approximation and the numerical calculation, 2 corresponds to 1%, and so on. We see that for the zeroth order approximation (without the $1.16\beta^{1/3}$) our approximation is good to within less than 10% error for *N/S* greater than ~ 100 and *E/N* greater than ~ 25. With the first order correction, the approximation is good to within less than 10% for *N/S* greater than ~ 3 and *E/N* greater than ~ 5.

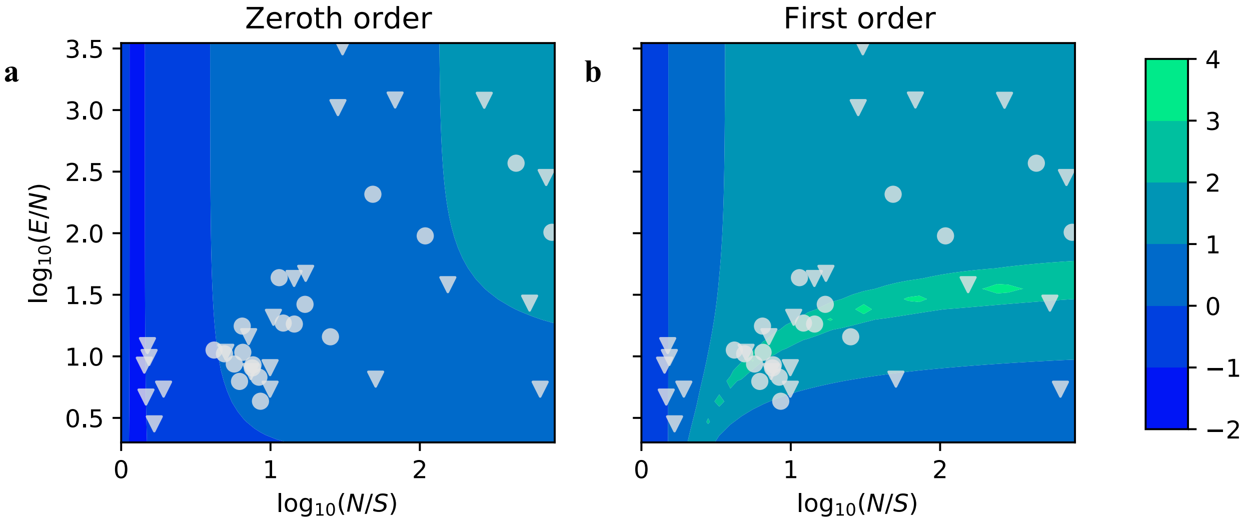

Fig. S2. Contour plots showing the accuracy of our analytic approximation for calculating biomass. The contour lines show the number of decimal places that are accurate between the zeroth order (Fig. S2**a**) or first order (Fig. S2**b**) approximations, and the exactly calculated numerical result. This is calculated as $-\log_{10}\left|\frac{B_{\text{approx}}-B_{\text{num}}}{B_{\text{num}}}\right|$, where B_{approx} is the biomass calculated using the analytic approximation, and B_{num} is calculated numerically. The axes correspond to varying E and N with S held constant at 50, and points correspond to the empirical data in Table 1. Note that S can be held constant because B/S depends only on E/S, and the constraints depend only on E/S and N/S, when S is large enough. However, for small S in this range, N can also be quite small, and we see the effects of the finite sum when calculating the constraints. This means that we must take S large enough that we do not see these effects, which in practice is about $S = 50$. We have therefore highlighted the data points where $S < 50$ by marking them with a downward triangle rather than a circle to show that the indicated accuracy of the approximation may not be correct for these points, though we note that in practice it is quite similar.

Supplementary Note 3: Data sources and extended acknowledgements

Data sources

A.

Data source and taxa	Plot	Year	Area (ha)	*S*	*N*	*E*	*B*
Tropical trees							
CSIRO permanent rainforest plots of North Queensland (Queensland, Australia) (S3)	EP3	1973	0.5	67	506	4074.955	11131.86
	EP18	1975	0.5	79	452	3915.177	11332.63
	EP19	1977	0.5	64	397	2482.458	6218.057
	EP29	1981	0.5	49	487	2634.251	5684.631
	EP30	1976	0.5	66	552	3737.412	9528.809
	EP31	1978	0.5	47	236	2544.609	8096.39
	EP32	1983	0.5	51	437	1887.393	4519.255
	EP33	1990	0.5	43	307	4435.325	15840.59
	EP34	1984	0.5	59	290	3040.91	9044.354
	EP40	1998	0.5	48	477	3859.423	11095.37
	EP41	1983	0.5	50	369	2775.138	6739.585
	EP42	1977	0.5	58	243	2732.479	9317.053
	EP43	1984	0.5	59	385	4151.287	12856.02
	EP44	1990	0.5	58	443	3779.391	10811.51
CTFS (Panama)	BCI (S4-S6)	1982	50	307	235338	23944860	405723649
	Cocoli (S7)	1997	2	171	8290	1717206	29636476
	Sherman (S7)	1997	5.96	225	24453	2325982	24937441
Catuba (Acre, Brazil) (S8)	Catuba	1999	20	156	1009	17760.1	62397.22

Atlantic Forest restinga (Espírito Santo, Brazil) (S9)	6L1	2015	0.00125	15	22	102.7092	224.3061
	6L3	2015	0.00125	14	20	169.6116	568.2694
	5L1	2015	0.00125	13	25	135.3977	371.2233
	5L2	2015	0.00125	13	20	195.7322	627.4894
	5L3	2015	0.00125	15	25	70.73095	110.6893
	5L5	2015	0.00125	18	27	329.9215	1383.537

B.

**Data source and taxa**	**Plot**	**Year**	**Area (ha)**	***S***	***N***	***E***	***B***
Temperate trees							
Harvard Forest (Massachusetts, USA)	EMS Tower (S10)	1993	1.26	15	759	4953.7	10880.94
	Lyford mapped tree plot (S11)	1969	2.88	24	3696	141101	655151.6
Hubbard Brook Experimental Forest (New Hampshire, USA) (S12)	Watershed 6	2017	13.23	16	10230	55324	117597.1
Kellogg Biological Station (Michigan, USA) (S13)	Deciduous Forest 1	2018	0.81 (*)	18	259	11105	57231.16
	Deciduous Forest 2	2018	0.5 (*)	10	172	8115.8	43636.65
	Deciduous Forest 3	2018	0.35 (*)	13	136	2820.1	10936.14
SCBI Large Forest Dynamics Plot (Virginia, USA) (S14)	Front Royal	2012	25.6	68	29986	11102334	153035741
Ordway Swisher Forest Dynamics Plot (Florida, USA) (S15)	unburned	2019	17.6	11	7714	2185400	18386158
Traunstein Forest Dynamics Plot (Germany) (S16)	CTFS	2016	25	29	15758	425994	1679991

C.

**Data source and taxa**	**Plot**	**Year**	**Area (ha)**	***S***	***N***	***E***	***B***
Temperate forest communities							
UC Santa Cruz (California, USA) (S17)	FERC	2006	6	31	8370	10046216	223493837
Point Reyes National Seashore Bishop Pines (California, USA) (S18)	Mt. Vision	2012	0.0256	27	1844	2223879 (*)	123940275
	Bayview	2012	0.0256	16	486	1585384 (*)	38793345
Subalpine meadow vascular plants							
Rocky Mountain Biological Laboratory (Colorado, USA) (S19)	Belleview	2012	0.0064	31	877	917872	17532137

D.

**Site**	**Plot**	**Year**	***S***	***N***	***B***	***E***
Tropical island arthropods Hawaii, USA (S20)	Volcano (200 y)	1997	167	1909	424260.2	83121.76
	Lanai (1500 y)	1997	156	2253	165838.1	41210.11
	Kohala (150 ky)	1997	240	6048	339471.8	87168.36
	Molokai (1.2 My)	1997	227	3865	413508.2	102189.2
	Kauai (4My)	1997	158	1922	137916.7	35818.52

Table S2. Sources of data and empirical values of state variables for the data used to test the Ecological Equation of State. Tables show values associated with Tropical trees (A), Temperate trees (B), Vascular plant communities (C), and Tropical island arthropods (D). "Year" denotes year the survey was started. Area is reported in hectares (ha). For Hawaii arthropods, the

geologic age of each site is given in parentheses and sampling was carried out by fumigating tree canopies of varying areas. Plant size data were converted to metabolic rates and rescaled such that $\boldsymbol{\varepsilon_{min}} = \mathbf{1}$; rescaled metabolic rate measurements of individuals were summed to calculate *E*; and $B = \sum_1^N metabolic\ rate_{i,rescaled}{}^{4/3}$. For animal data, mass measurements directly measure biomass, and therefore the metabolic rates are the calculated quantities. The individual with the smallest mass was therefore rescaled to $\varepsilon_{min} = 1$; and rescaled masses were then summed to calculate *B*, and *E* was calculated as $E = \sum_1^N mass_{i,rescaled}{}^{3/4}$. The symbol (*) indicates an estimated value. All reference numbers in the Table refer to the Main Text reference list.

Extended acknowledgements

We thank Point Reyes National Seashore for providing permits, field sites, logistical support, and facilities. Data were collected under PRNS Park-assigned permit PORE-2012-SCI-0014, Activity #PORE-00572. We also thank the Rocky Mountain Biological Laboratory for providing permitting, logistical support, and facilities, and Gunnison National Forest for providing field sites. These datasets were collected by EAN and others with funding provided by the NSF in the form of the Graduate Research Fellowship Program, Research Experience for Undergraduates program, and grant NSF-EF-1137685.

We thank Dr. Dan Gruner for sharing Hawaii arthropod data with us directly.

We thank the Harvard Forest for making available tree plot datasets under Creative Commons CC0 1.0 licenses (No Rights Reserved).

We thank the Commonwealth Scientific and Industrial Research Organization of Australia for making the data from the CSIRO permanent rainforest plots of North Queensland available under a Creative Commons International license (CC BY 4.0; https://creativecommons.org/licenses/by/4.0/).

Data for the Catuaba, Brazil forest plot were made available through the Oak Ridge National Laboratory Distributed Active Archive Center (ORNL DAAC), which is a NASA Earth Observing System Data and Information System (EOSDIS) data center. Data were accessed in October of 2021, at https://daac.ornl.gov/LBA/guides/LC02_PermPlot_Acre.html. We thank ORNL DAAC, the data collectors, and the data managers.

We thank the Environmental Data Initiative for the datasets from Hubbard Brook Experimental Forest (data package ID: knb-lter-hbr.239.1; Creative Commons license CC BY 4.0); and from Kellogg Biological Station (KBS). We additionally thank Drs. G. Philip Robertson, Sven Böhm, and Nick Haddad for granting permissions for KBS data use directly. Data use from KBS is made possible by support from the NSF through the Long-Term Ecological Research (LTER) program.

We thank the Center for Tropical Forest Science (CTFS) of the Smithsonian Tropical Research Institute and ForestGeo for datasets from Traunstein Forest Dynamics Plot, UC Santa Cruz FERC, SCBI Large Forest Dynamics Plot, Ordway Swisher Forest Dynamics Plot, Cocoli, Sherman, and Barro Colorado Island (BCI).

The Traunstein Forest Dynamics Plot is a collaborative project of the Chair for Forest Growth and Yield, Technische Universität München, the Department of Ecological Modelling at the Helmholtz Centre for Environmental Research UFZ, Leipzig, and the Microwaves and Radar Institute at the German Aerospace Center - DLR, Oberpfaffenhofen. Plot establishment and the first survey have been funded by the Helmholtz Research Alliance “Remote Sensing and Earth System Dynamics”. We thank the Municipal Forest Administration of the City of Traunstein for making this project possible on their estate property.

We thank Dr. Dan Johnson for permissions and extra data preparation for the Ordway Swisher plot. The Ordway Swisher Forest Dynamics Plot (OSFDP) is supported by the University of Florida Institute of Food and Agricultural Sciences (IFAS). Funding was provided by the IFAS Ordway Swisher Jumpstart Award. Support was provided by the USDA National Institute of Food and Agriculture McIntire-Stennis projects1007080 and 1018790. We thank the Ordway Swisher Biological Station staff for providing logistical support, and many students and researchers that have contributed to the project.

The University of California Santa Cruz (UCSC) Forest Ecology Research Plot was made possible by National Science Foundation grants to Gregory S. Gilbert (DEB-0515520 and DEB-084259), by the Pepper-Giberson Chair Fund, the University of California, and the hard work of dozens of UCSC students. The plot project is part the Forest Global Earth Observatory (ForestGEO), a global network of large-scale demographic tree plots.

We thank Dr. Jehová Lourenço, Jr. and multiple scientists and technicians for data from the Brazilian Atlantic Forest restinga plots, including Camilla Rozindo Diaz Milanez, Luciana Dias Thomaz, Douglas Tinoco Wandekoken, Felipe Barreto, Fabiano Volponi, Jocimara S. P. Lourenço, Nilton E. Oliveira Filho, Rodrigo Theófilo, José M. L. Gomes, and Danielly Hirata.

The BCI forest dynamics research project was founded by S.P. Hubbell and R.B. Foster, and is now managed by R. Condit, S. Lao, and R. Perez under the Center for Tropical Forest Science and the Smithsonian Tropical Research in Panama. Numerous organizations have provided funding, principally the U.S. National Science Foundation, and hundreds of field workers have contributed.

Supplementary Note 4: Sensitivity of the accuracy of the equation of state to the metabolic scaling law

In the main text, the ecological equation of state is derived under the assumption that the metabolic rate of individual organisms scale as mass to the 3/4 power. Although there is considerable evidence for such a relationship, alternative scaling exponents ranging from as low as 2/3 to 1 or higher have been proposed, depending upon type of taxonomic group and age of a cohort. The value 2/3 is motivated by a simple surface-to-volume argument.

Here we investigate the sensitivity of the equation of state to the choice of scaling exponent. As in Supplementary Note 1, we let σ be the inverse of the scaling exponent and then using $B = S\sum_n \int d\varepsilon\ n\varepsilon^{\sigma} R(n,\varepsilon|S,N,E)$, for $\sigma > 1$ we derive:

$$B \approx \frac{\Gamma(\sigma+2)}{2(\sigma-1)} \frac{E^{\sigma}}{S^{\sigma-1}\ln\left(\frac{1}{\beta}\right)} \qquad (\sigma > 1) \tag{S7}$$

where β is calculated from $\beta\ln(1/\beta) \approx S/N$. For the special case of $\sigma = 1$ we derive:

$$B = E \qquad (\sigma = 1). \tag{S8}$$

That $B = E$ if $\sigma = 1$ can be seen from the fact that one of the METE constraint conditions is $E = S\sum_n \int d\varepsilon\ n\varepsilon\ R(n,\varepsilon|S,N,E)$.

Because both mass and metabolic rate of all individuals are rarely measured, comparison of the above predictions with empirical data, requires estimating observed metabolic rate from measured mass, or vice versa. If masses are measured, then metabolic rates are calculated from $\varepsilon \sim m^{1/\sigma}$, while if metabolic rate is measured, then mass is computed using $m \sim \varepsilon^{\sigma}$. For trees, we will continue to assume that metabolic rate scales isometrically with basal area.

We first observe that if $\sigma = 1$ is assumed, then the equation of state, $B = E$, is an identity and the observed B will always equal the predicted B. To see this, assume that metabolic rate is measured and the empirical mass of each individual is calculated, using $\sigma = 1$, to be equal to the metabolic rate. Or equivalently, if mass is measured, then the empirical metabolic rate of each individual is calculated to equal its mass. In both cases, $B = E$. Parenthetically, we note that another trivial

result, this time holding for any σ, is that if all individuals have the same measured metabolic rate, then in our units the metabolic rate values are 1 for all individuals, resulting again in a $B = E$ equation of state.

We next compare the validity of the equation of state for the two scaling exponents 2/3 and 3/4. Comparing the same style figures in the main text, the prediction of B value by the 3/4 law is more accurate than the 2/3 law (Fig. S3 and Fig. 1). We also note that the 3/4 law predicts more of the variance in the ratio of $E: B^{1/\sigma}$ than does the 2/3 law (Fig. S4 and Fig. 2). In both results, the R^2 values of the simple regression of the 2/3 case are smaller than those of the 3/4 case.

Although the equation of state becomes an identity if $\sigma = 1$, it was not assured that the equation of state with a 3/4 scaling rule would outperform that with a 2/3 scaling rule under the assumption that our METE starting point is correct. If the true scaling exponent was exactly 2/3, then the equation of state derived under that condition might have outperformed the 3/4 result. For the data sets considered here, and under the assumption that the METE structure function, R, is valid, it thus appears more plausible to choose 3/4, rather than 2/3, metabolic scaling of biomass for both practical and empirical reasons: it predicts an equation of state relationship among the four state variables with high accuracy and is consistent with a model of the physiological features of the vascular system of plants and animals.

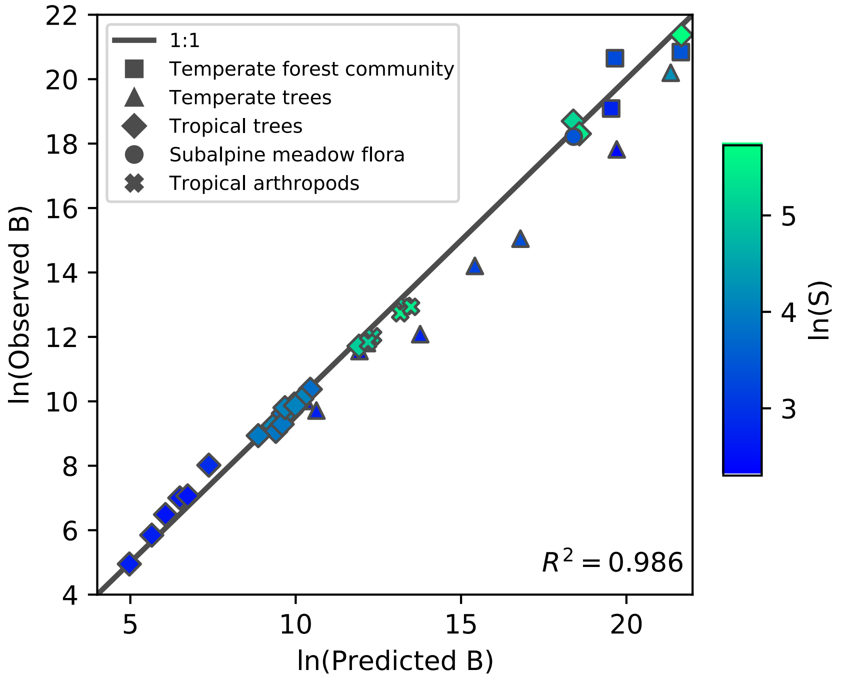

Fig. S3. Comparing observed and predicted values of *B* of each site. The same style plots as Fig. 1 in the main text is shown but here a different scaling relationship is used between metabolic rate $\boldsymbol{\varepsilon}$ and biomass $\boldsymbol{m}$ of an individual, $\boldsymbol{\varepsilon \sim m^{2/3}}$, namely $\boldsymbol{\sigma = 3/2}$. Lighter color corresponds to higher species richness.

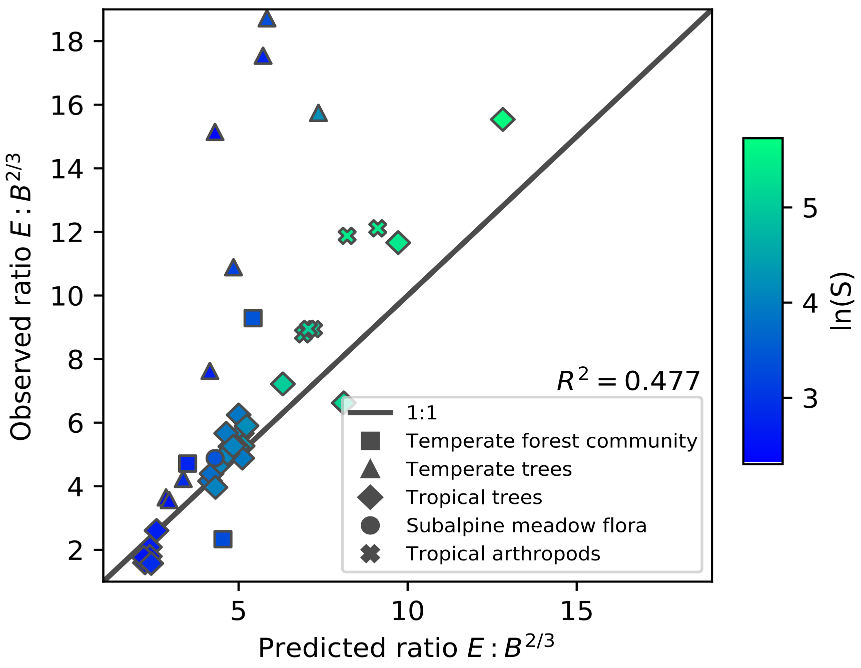

Fig. S4. Comparing observed and predicted values of the ratio of E and $B^{1/\sigma}$ of each site. The same style plots as Fig. 2 in the main text is shown but here a different scaling relationship is used between metabolic rate $\boldsymbol{\varepsilon}$ and biomass $\boldsymbol{m}$ of an individual, $\boldsymbol{\varepsilon \sim m^{2/3}}$, namely $\boldsymbol{\sigma = 3/2}$. Lighter color corresponds to higher species richness.

Supplementary References

S1. Harte, J., Umemura, K. Brush, M. DynaMETE: a hybrid MaxEnt-plus-mechanism theory of dynamic macroecology. *Ecol. Lett*. **24**, 935–949 (2021).

S2. Olver, F. W. J., Olde Daalhuis, A. B., Lozier, D. W., Schneider, B. I., Boisvert, R. F., Clark, C. W., Miller, B. R., Saunders, B. V., Cohl, H. S., McClain, M. A., eds., NIST Digital Library of Mathematical Functions. http://dlmf.nist.gov/8.19.E25, Release 1.0.28 of 2020-09-15.

S3. Bradford, M. G., Murphy, H., Ford, A., Hogan, D., Metcalfe, D. CSIRO Permanent Rainforest Plots of North Queensland. v3. (2014). CSIRO. Data Collection. https://doi.org/10.4225/08/59475c67be7a4

S4. Hubbell, S. P., Foster, R. B., O'Brien, S. T., Harms, K. E., Condit, R., Wechsler, B., Wright, S. J., de Lao S. L. Light-Gap disturbances, recruitment limitation, and tree diversity in a neotropical forest. *Science* **283**, 554–557 (1999).

S5. Condit, R. Tropical Forest Census Plots: Methods and Results from Barro Colorado Island, Panama and a Comparison with Other Plots. Springer Science & Business Media (1998).

S6. Condit, R. Pérez, R., Aguilar, S., Lao, S., Foster, R., Hubbell, S. Complete data from the Barro Colorado 50-ha plot: 423617 trees, 35 years. URL https://doi.org/10.15146/5xcp-0d46 (2019).

S7. Condit, R., Aguilar, S., Hernandez, A., Perez, R., Lao, S., Angehr, G., Hubbell, S. P., Foster, R. B. Tropical forest dynamics across a rainfall gradient and the impact of an El Niño dry season. *J. Trop. Ecol*. **20**, 51–72 (2004).

S8. Selhorst, D., Brown, I. F.. LBA-ECO LC-02 Biophysical Measurements of Forests, Acre, Brazil: 1999-2002. ORNL DAAC (2014) (https://daac.ornl.gov/cgi-bin/download.pl?ds_id=1237&source=schema_org_metadata).

S9. Lourenço Jr. J., Newman E. A., Ventura J. A., Milanez C. R., Thomaz L. D., Wandekoken D. T., Enquist B. J. Soil-associated drivers of plant traits and functional composition in Atlantic Forest coastal tree communities. *Ecosphere* **12(7)**: e03629 (2021).

S10. Munger, W., Wofsy, S. Biomass Inventories at Harvard Forest EMS Tower since 1993. Harvard Forest Data Archive: HF069 (v.36). (2021). Environmental Data Initiative: https://doi.org/10.6073/pasta/5c2f17c295413da2a2a091fd7696af40.

S11. Foster, D., Barker Plotkin, A. A., Lyford, W. Lyford Mapped Tree Plot at Harvard Forest since 1969. Harvard Forest Data Archive: HF032 (v.20). (2017). Environmental Data Initiative: https://doi.org/10.6073/pasta/292e47940d0b0b07c3be7e9026c12c66.

S12. Battles, J. J., Cleavitt, N., Johnson, C., Hamburg, S., Fahey, T., Driscoll, C., Likens, G. Forest Inventory of a Northern Hardwood Forest: Watershed 6, 2017, Hubbard Brook Experimental Forest ver 1. Environmental Data Initiative (2019). (https://doi.org/10.6073/pasta/0593ba15fb76a4f085797126a1bea3a7 (Accessed 2021-10-02).

S13. Robertson, G. P., Hamilton, S. K. Long-term ecological research at the Kellogg Biological Station LTER site. The ecology of agricultural landscapes: Long-term research on the path to sustainability, 1–32 (2015).

S14. Bourg, N. A., McShea, W. J., Thompson, J. R., McGarvey, J. C., Shen, X. Initial census, woody seedling, seed rain, and stand structure data for the SCBI SIGEO Large Forest Dynamics Plot. *Ecology*. **94**, 2111–2112 (2013).

S15. Johnson, D. J., Magee, L., Pandit, K., Bourdon, J., Broadbent, E., Glenn, K., Kaddoura, Y., Machado, S., Nieves, J., Wilkinson, B., Zambrano, A., Bohlman, S. Canopy tree density and species influence tree regeneration patterns and woody species diversity in a longleaf pine forest. *Forest Ecol. and Manag.***490**, 119082 ISSN 0378-1127 (2021).

S16. Pretzsch, H. "Diversity and productivity in forests: Evidence from long-term experimental plots", pp. 41-64 in *Forest Diversity and Function: Temperate and Boreal Systems*, Scherer-Lorenzen, M. ,Körner, C., Schulze, E.-D., Eds., Springer Berlin, Heidelberg (2005).

S17. Gilbert, G. S., Howard, E., Ayala-Orozco, B., Bonilla-Moheno, M., Cummings, J., Langridge, S., Parker, I. M., Pasari, J., Schweizer, D., Swope, S. Beyond the tropics: forest structure in a temperate forest mapped plot. *J. Veg. Sci*. **21**, 388–405 (2010).

S18. Newman, E. A., Wilber, M. Q., Kopper, K. E., Moritz, M. A., Falk, D., McKenzie, D., Harte, J. Disturbance macroecology: a comparative study of community structure metrics in a high-severity disturbance regime, Ecosphere **11**(1), e03022 (2020).

S19. Newman, E. A., Harte, M. E., Lowell, N., Wilber, M., Harte, J. Empirical tests of within- and across-species energetics in a diverse plant community. *Ecology* **95**(10), 2815-2825 (2014).

S20. Gruner, D. S. Geological age, ecosystem development, and local resource constraints on arthropod community structure in the Hawaiian Islands. *Biol. J. Linn. Soc. Lond*. **90**, 551–570 (2007).
