# Supplementary material for: An equation of state unifies diversity, productivity, abundance and biomass
Source: Commun Biol. 2022 Aug 25;5:874. doi: 10.1038/s42003-022-03817-8 (PMC9411111; doi:10.1038/s42003-022-03817-8)
Supplement: Supplementary file 3 — Description of Additional Supplementary Files [file 42003_2022_3817_MOESM3_ESM.pdf]

Description of Additional Supplementary Files

File name: Supplementary Video 1
Description: A 3D visualization of the state variables S, N, and E for all data analyzed. Each data point arranged in a 3D space of the logs of the state variables. If there were an obvious linear relationship among any of these variables, we would see the data collapse onto a straight line when viewed along a specific direction. Given that we do not see such a collapse, we assume that S, N, and E are only loosely related, and we require an additional state variable in order to derive an equation of state.
